# The Diagnostic and Grading Accuracy of ^68^Ga-DOTATATE and ^18^F-FDG PET/MR for Pancreatic Neuroendocrine Neoplasms

**DOI:** 10.3389/fonc.2022.796391

**Published:** 2022-02-22

**Authors:** Jinxin Zhou, Runze Zhao, Yu Pan, Huijun Ju, Xinyun Huang, Yu Jiang, Jiabin Jin, Yifan Zhang

**Affiliations:** ^1^ Department of Nuclear Medicine, Ruijin Hospital, Shanghai Jiao Tong University School of Medicine, Shanghai, China; ^2^ Department of General Surgery, Pancreatic Disease Center, Ruijin Hospital, Shanghai Jiao Tong University School of Medicine, Shanghai, China

**Keywords:** pancreatic neuroendocrine neoplasm, grading, ^68^Ga-DOTATATE, ^18^F-FDG, apparent diffusion coefficient, PET/MR

## Abstract

Accurate diagnosis and grading are critical for pancreatic neuroendocrine neoplasm (pNEN) management. This study compares the diagnostic and grading value of ^68^Ga-DOTATATE PET/MR and ^18^F-FDG PET/MR for pNENs separately as well as in combination. A total of 36 patients with histologically confirmed pNENs, who underwent both ^68^Ga-DOTATATE PET/MR and ^18^F-FDG PET/MR within 2 weeks from 2020 to 2021, were retrospectively collected and analyzed. The maximum standardized uptake values of ^68^Ga-DOTATATE (G-SUVmax) and ^18^F-FDG (F-SUVmax) on PET and the minimum values of apparent diffusion coefficient (ADCmin) on MR were measured on the lesions with known histological grading (25 by surgery, 11 by biopsy). Receiver-operating characteristic analysis was applied to determine the cutoffs of these parameters or their combinations for differentiation between G1 and G2, as well as between low-grade and high-grade pNENs. The Spearman rank correlation coefficient was used to assess the correlation between the imaging parameters and the maximum tumor diameters. The detection rate of ^68^Ga-DOTATATE PET imaging alone was 95%, 87.5%, and 37.5% for G1, G2, and G3, respectively. Adding ^18^F-FDG PET or MR sequences of PET/MR increased the detection rate to 100% in all grades. Among the three parameters, G-SUVmax had the highest diagnostic rate in predicting tumor grade. It presented a sensitivity of 87.5% and a specificity of 80.0% with a cutoff value of 42.75 for differentiating G2 from G1 pNETs and a sensitivity and specificity of 100% and 71.4% with a cutoff value of 32.75 in predicting high-grade pNENs. The ratio of G-SUVmax to F-SUVmax (G-SUVmax/F-SUVmax) showed slight improvement in the diagnostic rate, while the product of G-SUVmax and ADCmin (G-SUVmax*ADCmin) did not improve the diagnostic rate. ^68^Ga-DOTATATE PET/MR alone is sufficient for the diagnosis of pNENs and the prediction of various grades.

## Introduction

Pancreatic neuroendocrine neoplasms (pNENs) are rare, but their incidence has been increasing in the recent years with an estimated annual incidence of approximately 0.5/100,000, accounting for 10% of all neuroendocrine neoplasms (NENs) (1, 2). Over 70% of pNENs are non-functional, while the most common functional pNEN is islet cell tumor, followed by gastrinoma (3–6). Most pNENs have malignant manifestations, and more than 60% present with metastasis at diagnosis (7–9). Despite high metastatic rates, the prognosis of pNENs is much more favorable than that of pancreatic adenocarcinoma, with a median overall survival of more than 5 years. The 20-year disease-specific survival rate of patients without metastasis can be up to 50% after radical resection (2, 10).

Neuroendocrine neoplasms (NENs) can be grouped into grades of G1, G2, and G3 according to mitotic count and Ki-67 index. In G1 tumors, the mitotic count is <2 cells/2 mm^2^ and the Ki-67 index is <3%; in G2 tumors, the mitotic count is 2–20 cells/2 mm^2^, and the Ki-67 index is 3%–20%; and in G3 tumors, the mitotic count is >20 cells/2 mm^2^ and the Ki-67 index is >20% (11). Pathologically, G1 is low grade, G2 is medium grade, and G3 is high grade. The higher the pathological grade, the worse the prognosis of the patient (12). Similarly, pNENs are a heterogeneous group of malignancies that can also be graded from G1 to G3, or simply divided into well-differentiated and poorly differentiated types (12).

Accurate pathological grading is of great value in the selection of a treatment scheme, prediction of prognosis, and follow-up. However, due to the heterogeneity of pNENs, tumor grading may not be uniform within a lesion or among different lesions in the same patient. Therefore, local puncture biopsies and surgical pathology of a section of the lesion may underestimate or overestimate the grading (13). Therefore, systemic functional imaging techniques targeting pNENs may be more suited for comprehensive grading. Studies have shown that 80%–100% of pNENs express a somatostatin receptor (SSTR) on the surface of cells, mainly type 2 SSTR (14). At present, the most frequently used SSTR-based functional imaging technique is positron emission computed tomography (PET) with ^68^gallium-labeled-somatostatin analog (SSA) (^68^Ga-DOTA-SSA), including ^68^Ga-DOTATOC, ^68^Ga-DOTATATE, and ^68^Ga-DOTANOC, with almost the same diagnostic efficacies (15, 16). A meta-analysis showed that the sensitivity and specificity of ^68^Ga-DOTA-SSA PET or PET/CT in the diagnosis of NENs are 93% and 91%, respectively (17). The maximum standardized uptake value (SUVmax) of ^68^Ga-DOTA-SSA PET/CT imaging has been reported to correlate with the pathological grade of gastro-entero-pancreatic (GEP) NENs. The Ki-67 index was reported to negatively correlate with SUVmax, while there was no correlation between mitotic count and SUVmax in the GEP NENs (18). To our knowledge, studies specifically focusing on these imaging techniques in pNENs are lacking.

Fluorodeoxyglucose (FDG) PET is widely used in malignant tumor imaging. Most malignant tumors have increased glucose metabolism presenting as high FDG uptake. Although ^18^F-FDG PET is not recommended for routine use in NENs, its application value in high-grade NENs (G3 NETs) has been gradually recognized (19, 20). Studies have shown that the sensitivity of ^18^F-FDG PET/CT imaging for G1/G2 GEP-NETs is only 40%–60%, but for G3 tumors it can be up to 95% (21, 22). Kayani et al. reported that ^18^F-FDG PET/CT and ^68^Ga-DOTATATE PET/CT alone each have a sensitivity of 66% and 82%, respectively, while ^18^F-FDG and ^68^Ga-DOTATATE dual-tracer PET/CTs have an increased sensitivity of 92% (21). A higher ^18^F-FDG uptake often indicates higher invasiveness and worse prognosis of NENs, and the combination of ^68^Ga-DOTA-SSA and ^18^F-FDG PET/CT imaging could improve prognosis predictions for NEN patients beyond that of the pathological grading system using WHO 2010 standard (23–25).

^68^Ga-DOTA-SSA PET/MR can detect more lesions than PET/CT, mainly due to the higher sensitivity of MR sequences in lesion detection than localizing CT (26). The advantages of MR sequences also help with preoperative grading. The apparent diffusion coefficient (ADC) value in the MR diffusion sequence has a reverse correlation with the pathological grading of GEP NENs: the higher the pathological grade, the lower the ADC value. The minimum ADC value (ADCmin) has the most significant value (27–29).

Recognizing the value of ^18^F-FDG PET/CT in the diagnosis and classification of NENs, other multimodal imaging techniques have been developed for clinical application, in addition to the original ^68^Ga-DOTA-SSA PET/CT imaging. However, there is still the important dilemma with balancing improved diagnostic efficiency with increased imaging cost and radiation exposure related to additional procedures. Therefore, the current study intends to determine the best imaging modality specifically for pNEN patients by comparing the diagnostic and grading efficiencies of various parameters of ^68^Ga-DOTATATE and ^18^F-FDG PET/MR, used either separately or in combination.

## Materials and Methods

### Basic Information of Patients and Lesions

This study retrospectively retrieved 100 patients who underwent both ^68^Ga-DOTATATE and ^18^F-FDG PET/MR imaging in our center for evaluation of pancreatic or pancreatic-derived lesions from March 1, 2020, to June 20, 2021. This study was approved by the ethics committee of our hospital (IRB approval number: 2020-52), and all patients provided informed consent.

**Figure 1:**
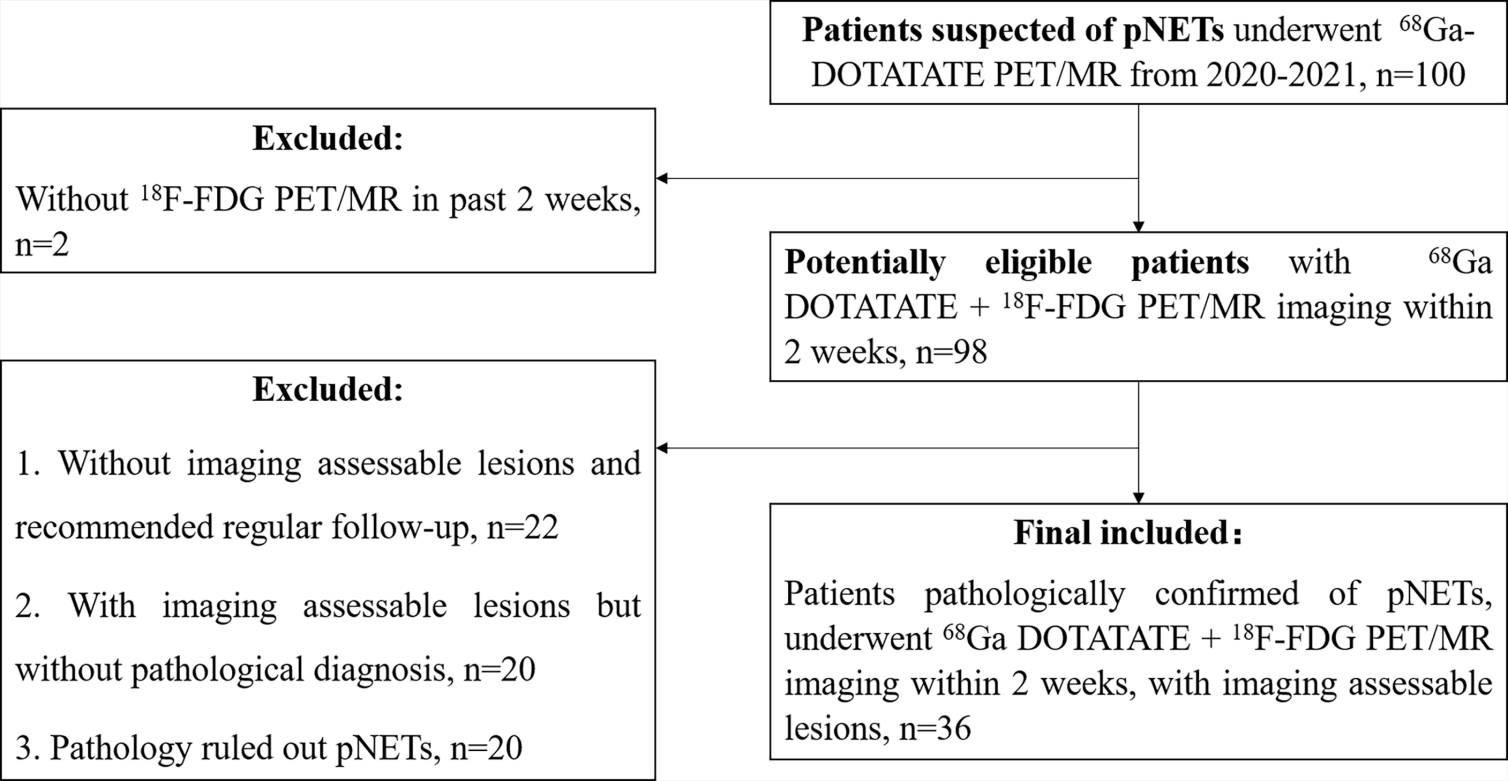
Flowchart.

The inclusion criteria were as follows: (1) ^68^Ga-DOTATATE PET/MR imaging was performed for suspected pancreatic or pancreatic-derived neuroendocrine tumors. (2) The DWI/ADC sequence was included in PET/MR imaging protocol. (3) The interval between ^68^Ga-DOTATATE PET/MR and ^18^F-FDG PET/MR was less than 2 weeks. (4) There were positive lesions on at least one imaging. (5) After imaging, the target lesion was pathologically diagnosed as pancreatic neuroendocrine tumor or its metastasis. (6) If multiple lesions with pathological results were present, the largest lesion in the primary or metastatic sites was selected for analysis (**Figure 1**). In total, 36 patients met the inclusion criteria and were enrolled in this study, with a median age of 50 years and an age range of 22–75 years, including 11 males and 25 females (**Table 1**).

**Table 1:** Patient and tumor characteristics.

Characteristic	Data
**Age**	50 [41.5, 59]
**Gender**	
** Male**	11 (30.6%)
** Female**	25 (69.4%)
**Histologic tumor grade**	
** G1**	20 (55.6%)
** G2**	8 (22.2%)
** G3+NEC**	8 (22.2%)
**Pathological sources**	
** Surgery**	25 (69.4%)
** Biopsy**	11 (30.6%)
**Location**	
** Pancreas**	22 (61.1%)
** Liver**	7 (19.4%)
** Lymph node**	7 (19.4%)

Continuous data are expressed as median [25%_L_,75%_U_]. Qualitative data are expressed as numbers followed by percentages in parentheses.

### ^68^Ga-DOTATATE and ^18^F-FDG PET/MR Imaging Procedure

^68^Ga-DOTATATE PET/MR and ^18^F-FDG PET/MR were completed on two different days, and the intervals between the two examinations were within 2 weeks. The ^18^F-FDG tracer was synthesized automatically using the tracer synthesis system of the TRACERlab FXF-N (GE Healthcare, Chicago, IL, USA), with a radiochemical purity >95%. Before ^18^F-FDG examination, patients were required to fast for at least 6 h and the level of fasting blood glucose should be less than 11.1 mmol/l. For patients with diabetes, insulin should not be used on the day of ^18^F-FDG examination. Whole-body PET/MR imaging was performed 45–90 min after intravenous injection of 2–5 MBq/kg ^18^F-FDG. For ^68^Ga-DOTATATE PET/MR, octreotide therapy was not required to terminate before ^68^Ga-DOTATATE examination (30–32). PET/MR imaging was performed 45–60 min after intravenous injection of 2 MBq/kg ^68^Ga-DOTATATE (total amount ≤ 200 MBq).

Whole-body PET/MR was performed using an integrated PET/MR system (Biograph mMR; Siemens Healthineers, Erlangen, Germany). The scanning range was from the top of the skull to 1/3 of the length down the upper thigh. ^18^F-FDG PET images of the head and the body were acquired separately. The acquisition time of head PET images was 8 min with one bed. The acquisition time of body images was 4 min/bed, and a total of 4–5 beds were acquired. ^68^Ga-DOTATATE PET images were acquired successively from the head down to the body with the upper limbs of the patients placed on both sides of the body. The acquisition time was 4 min/bed, and a total of 5–6 beds were acquired. PET data were reconstructed using a three-dimensional attenuation-weighted ordered-subset expectation maximization method (2 iteration, 21 subsets, 256 × 256 matrix) and a Gaussian smoothing kernel with full width at half maximum (FWHM) of 6 mm. MRI sequences were acquired simultaneously during PET image acquisition, including a cross-sectional T2-weighted 2D half-Fourier acquisition single-shot turbo spin-echo (HASTE) sequence, cross-sectional echo-planar diffusion-weighted imaging (DWI) sequence (b values were 50 and 800 s/mm^2^, respectively), cross-sectional T1-weighted imaging, and T1WI-Dixon sequence. The ADC value was calculated using single exponential function (b values of 50 and 800 s/mm^2^).

### Qualitative and Quantitative Analyses of PET/MR Imaging

The labeling of positive lesions on ^68^Ga-DOTATATE and ^18^F-FDG PET/MR images was completed by two qualified nuclear medicine doctors who worked separately and summarized by a third investigator. The two observers labeling lesions and the third investigator summarizing lesions had radiology and nuclear medicine working experience of 8, 20, and 4 years, respectively. The original images were exported in DICOM format and processed with RadiAnt DICOM Viewer software (version 2021.1, Medixant Company, Poznan, Poland). The observers were randomly distributed with DICOM data without medical history. Positivity of a lesion in ^68^Ga-DOTATATE and ^18^F-FDG PET was defined as intensity greater than expected surrounding physiologic uptake. The positive lesions in MR were labeled by experience. Consensus was achieved by discussion.

The target lesions were selected retrospectively. The surgically resected lesions were located according to surgical records based on the preoperative and postoperative imaging results. For the patients confirmed by pathological biopsy, the lesion where the needle was located on CT images was selected for analysis. When multiple lesions from one patient were resected, the largest lesion identified by imaging was selected for analysis. It was considered to be successfully detected when the target lesion was labeled by the doctors in every patient.

The measurement of imaging parameters was performed by the two nuclear medicine doctors who labeled the lesions. They delineated the region of interest (ROI) of each lesion by manually delineating the tumor boundary at the maximum section of the target lesion. The target lesions in negative images were marked by the third investigator by copying the ROI from the positive images. The maximum normalized uptake value (SUVmax) and the minimum apparent diffusion coefficient (ADCmin) in the ADC sequence on MR images were automatically calculated by the software.

Among the included lesions, 22 cases were located in the pancreas, with an average maximum diameter of 3.3 cm (range 1.0–13.8 cm); 7 cases were located in the liver, with an average maximum diameter of 2.2 cm (range 1.3–3.4 cm); and 7 cases were located in lymph nodes, with an average maximum short diameter of 4.1 cm (range 1.6–8.8 cm). All the target lesions were pathologically diagnosed by surgery (25 cases) or by biopsy (11 cases) as pancreatic neuroendocrine tumors or pancreatic-derived neuroendocrine tumor metastases. According to the WHO 2019 pathological classification standard of gastrointestinal neuroendocrine tumors, 20 patients had grade G1 tumors, 8 patients had grade G2 tumors, and 8 patients had grade G3 or above tumors.

### Statistical Analysis

Statistical analysis was performed using SPSS statistics (version 20; IBM, Armonk, NY, USA), and GraphPad Prism (version 20; GraphPad Software LLC, San Diego, CA, USA) was used to plot the results.

Continuous variables were presented as the median [25%_L_,75%_U_], while categorical data were expressed as numbers (percentage). Normality was tested using the Kolmogorov–Smirnov Test. Continuous variables were compared using the Mann–Whitney test (with two-tailed probability). Categorical variables were evaluated using the chi-square test. Spearman correlation coefficients were calculated to assess the direction and strength of correlation between 2 variables. Interpretation was as follows: a positive or negative correlation with a coefficient of 0.90–1.00 was considered very high; 0.70–0.89 was considered high; 0.40–0.69 was considered moderate; 0.30–0.49 was considered low; and 0–0.29 was considered negligible. Receiver-operating-characteristic (ROC) analysis was performed to establish cutoff values for differentiation. The diagnostic value was expressed by sensitivity and specificity.

*p* value < 0.05 indicated that the difference was statistically significant.

## Results

### Detection Rates of Different Grades of pNENs by ^68^Ga-DOTATATE and ^18^F-FDG PET/MR Imaging

The two nuclear medicine doctors independently interpreted the ^68^Ga-DOTATATE and ^18^F-FDG PET/MR images. All the target lesions at various grades were effectively labeled (**Table 2**).

**Table 2:** Detection rate of different grade pNETs.

Methods	Total DR (%)	G1-DR (%)	G2-DR (%)	G3-DR (%)
^68^Ga-DOTATATE PET only	80.6	95	87.5	37.5
^18^F-FDG PET only	72.2	50	100	100
^68^Ga-DOTATATE and ^18^F-FDG PET	100	100	100	100
^68^Ga-DOTATATE PET/MR	100	100	100	100

DR, detection rate.

The detection rate of ^68^Ga-DOTATATE PET alone in all target pNEN lesions and metastases included in this study was 80.6%. As expected, it showed a higher detection rate for G1 and G2 pNENs at 95% and 87.5%, respectively. However, the detection rate of ^68^Ga-DOTATATE PET alone decreased significantly for G3 pNENs, with a detection rate of only 37.5%. The detection rate of ^18^F-FDG PET imaging alone for all target lesions was 72.2% in this study, specifically 50% for G1 pNENs, but 100% for G2, G3, and above pNENs. ^68^Ga-DOTATATE PET/MR imaging (PET + MR) or ^68^Ga-DOTATATE PET combined with ^18^F-FDG PET imaging both can effectively detect various grades of target pNEN lesions, with detection rates of 100% in this study.

### G-SUVmax, F-SUVmax, and ADCmin of Three Grade pNENs

In ^68^Ga-DOTATATE PET imaging, the G-SUVmax of G1 pNEN was significantly higher than that of G2 pNENs [60.0 (46.25, 105.68) vs. 27.46 (21.74, 39.00), *p* < 0.01]. The G-SUVmax of G2 pNEN was also significantly higher than that of G3 and above pNENs [27.46 (21.74, 39.00) vs. 8.29 (5.47, 24.20), *p* < 0.05]. Meanwhile, the G-SUVmax was also significantly higher in low-grade (G1, G2) than in high-grade (G3, NEC) pNENs [49.99 (28.36, 77.63) vs. 8.29 (5.47, 24.20), *p* < 0.0001].

In ^18^F-FDG PET imaging, the F-SUVmax of G1 pNENs was significantly lower than that of G2 pNENs [2.44 (1.82, 3.61) vs. 5.75 (4.12, 11.08), *p* < 0.05]. The F-SUVmax of G3 and above pNENs was possibly higher than G2, but there were no significant differences. However, the F-SUVmax of high-grade pNENs was significantly higher than that of low-grade pNENs [11.18 (7.69, 14.19) vs. 3.04 (2.13, 6.12), *p* < 0.01].

**Figure 2:**
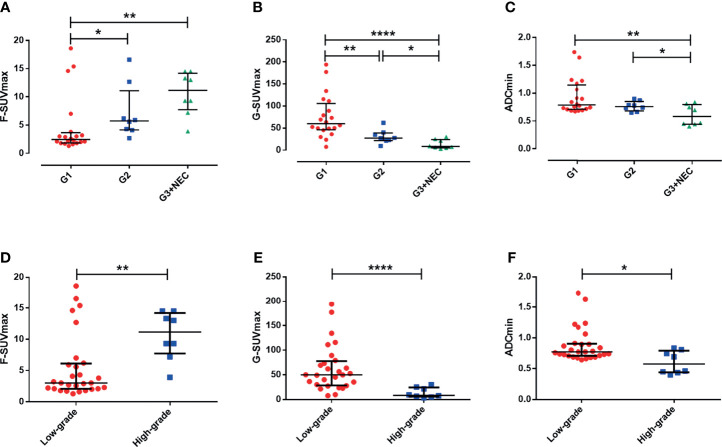
Differences of imaging parameters in various grades of pNENs. **(A)** The F-SUVmax of G2 and G3/NEC pNENs were significantly higher than that of G1, but F-SUVmax was not significantly different between G2 and G3/NEC pNENs. **(B)** G-SUVmax decreased gradually with the increase of pathological grade of the lesions, and there were significant statistical differences in G-SUVmax between G1, G2, and G3/NEC tumors. **(C)** The ADCmin of G3/NEC pNENs was significantly lower than that of G1 and G2 pNENs, but there was no significant difference in ADCmin between G1 and G2 pNENs. **(D)** The F-SUVmax of high-grade pNENs was significantly higher than that of low-grade pNENs. **(E)** The G-SUVmax of high-grade pNENs was significantly lower than that of low-grade pNENs. **(F)** The ADCmin of high-grade pNENs was significantly lower than that of low-grade pNENs. *p < 0.05, **p < 0.01, ****p < 0.0001.

In terms of ADCmin, there was no significant difference between G1 and G2 pNENs. However, the ADCmin of G1 and G2 pNENs were both significantly higher than that of G3 [G1: 0.79 (0.71, 1.159), G2: 0.76 (0.68, 0.85) G3: 0.58 (0.44, 0.79); G1 vs. G3 *p* < 0.01, G2 vs. G3 *p* < 0.05]. The ADCmin of high-grade pNENs was significantly lower than that of low-grade pNENs [0.58 (0.44, 0.79) vs. 0.77 (0.71,0.91), *p* < 0.05] (**Figure 2**, **Table 3**).

**Table 3:** Comparison of imaging parameters between different grade pNETs.

	G1 (n = 20)	G2 (n = 8)	G1 + G2 (n = 28)	G3 + NEC (n = 8)
**Diameters (cm]**	1.50 [1.23, 2.33]	2.45 [1.78, 7.60]	1.70 [1.20,2.58]	4.25 [3.00, 8.05]
**G-SUVmax**	60.0 [46.25, 105.68]	27.46 [21.74, 39.00]	49.99 [28.36, 77.63]	8.29 [5.47, 24.20]
**F-SUVmax**	2.44 [1.82, 3.61]	5.75 [4.12, 11.08]	3.04 [2.13, 6.12]	11.18 [7.69, 14.19]
**ADCmin [×10^-3^ mm^2^/s]**	0.79 [0.71, 1.15]	0.76 [0.68, 0.85]	0.77[0.71, 0.91]	0.58 [0.44, 0.79]
**G-SUVmax/F-SUVmax**	24.31 [9.95, 47.00]	4.10 [13.31, 6.41]	11.73 [3.96, 36.06]	0.86 [0.47, 2.40]
G-SUVmax*ADCmin	47.81 [33.81, 82.36]	21.03 [17.72, 28.78]	39.13 [21.83, 79.86]	4.34 [2.46, 18.12]

Data are median [25%_L_,75%_U_].

### The Values of G-SUVmax, F-SUVmax, and ADCmin in Distinguishing G1 and G2 pNENs

In order to determine the threshold values of G-SUVmax and F-SUVmax that could be used to distinguish G1 and G2 pNENs, we carried out ROC analysis and used the highest sum of sensitivity and specificity as the diagnostic threshold. When G-SUVmax < 42.75 was used as the criteria to diagnose G2 pNENs, the diagnostic efficiency was the highest, with a corresponding sensitivity of 87.5%, a specificity of 80.0%, and an AUC value of 0.85. When F-SUVmax > 3.95 was used as the threshold to diagnose G2 pNENs, the corresponding sensitivity and specificity were 87.5% and 80.0%, respectively. However, the AUC value was 0.79, which was lower than that of the G-SUVmax.

Since G-SUVmax negatively and F-SUVmax positively related to the grading of pNENs, the ratio of G-SUVmax and F-SUVmax (G-SUVmax/F-SUVmax) was introduced as an attempt to test its potential improvement in grading. The G-SUVmax/F-SUVmax ratio had similar sensitivity but slightly higher specificity than G-SUVmax alone in distinguishing G1 and G2 pNENs. The diagnostic efficiency was the highest when a G-SUVmax/F-SUVmax of 6.84 was used as the diagnostic threshold, with a corresponding sensitivity of 87.5%, a specificity of 85.0%, and an AUC value of 0.86.

**Figure 3:**
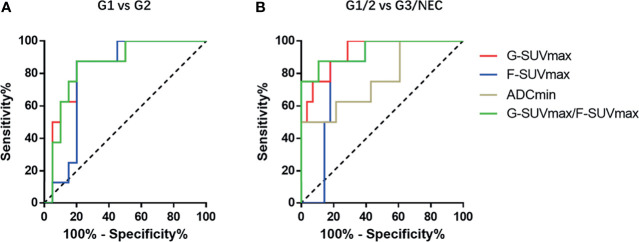
The ROC diagnostic efficiency curves of various imaging parameters in distinguishing G1 and G2 pNENs and differentiating low-grade and high-grade pNENs. **(A)** In distinguishing G1 and G2 pNENs, the diagnostic efficiency of G-SUVmax was higher than that of F-SUVmax, and the AUC value was 0.85. The diagnostic efficiency of G-SUVmax/F-SUVmax is comparable to that of G-SUVmax, and the AUC value was 0.86. **(B)** In distinguishing low-grade and high-grade pNENs, the diagnostic efficiency of G-SUVmax was also higher than that of F-SUVmax. The AUC value was 0.93, and the corresponding sensitivity and specificity were 100% and 71.4%, respectively. The diagnostic efficacy of G-SUVmax/F-SUVmax was roughly equivalent to that of G-SUVmax. The AUC value was 0.94, and the corresponding sensitivity and specificity were 87.5% and 89.3%, respectively.

G-SUVmax and ADCmin both were negatively related to grading, thus the product of G-SUVmax and ADCmin (G-SUVmax*ADCmin) was introduced as an attempt to test its potential improvement in grading. However, G-SUVmax*ADCmin did not improve the grading efficiency of pNENs in this study (**Figure 3**, **Table 4**).

**Table 4:** Comparison of cutoff values and diagnostic performance.

	G1 vs. G2, n = 28								G1/2 vs. G3/NEC, n = 36								
	AUC	Cutoff	SE,%	SP,%	TP	FP	FN	TN	AUC	Cutoff	SE,%	SP,%	TP		FP	FN	TN
G-SUVmax	0.85	42.75	87.5	80.0	7	1	4	16	0.93	32.75	100	71.4	8	0		8	20
F-SUVmax	0.79	3.95	87.5	80.0	7	1	4	16	0.81	7.1	87.5	82.1	7	1		5	23
ADCmin	NS	NS	NS	NS	NS	NS	NS	NS	0.77	0.70	62.5	78.6	5	3		6	22
G-SUVmax/ F-SUVmax	0.86	6.84	87.5	85.0	7	1	3	17	0.94	2.95	87.5	89.3	7	1		3	25
G-SUVmax* ADCmin	0.85	31.34	87.5	80.0	7	1	4	16	0.93	24.64	100	71.4	8	0		8	20

NS, not significant.

### The Values of G-SUVmax, F-SUVmax, and ADCmin in Distinguishing Low-Grade (G1, G2) and High-Grade (G3, NEC) pNENs

Regarding diagnosis and grading of high-grade pNENs, G-SUVmax showed the highest diagnostic efficiency. When G-SUVmax < 32.75 was used as the threshold, the diagnostic sensitivity was 100%, the specificity was 71.4%, and the AUC value was 0.93. When F-SUVmax > 7.1 was used as the threshold value, the diagnostic sensitivity was 87.5%, specificity was 82.1%, and the AUC value was 0.81. The diagnostic efficiency of ADCmin was the lowest. When ADCmin < 0.70 was used as the diagnostic threshold, the diagnostic sensitivity was 62.5%, the specificity was 78.6%, and the AUC value was 0.81.

The efficacy of the G-SUVmax/F-SUVmax ratio in distinguishing low-grade and high-grade pNENs was slightly higher than that of G-SUVmax alone. When 2.95 was used as the threshold value of G-SUVmax/F-SUVmax, the diagnostic sensitivity was 87.5%, the specificity was 89.3%, and the AUC value was 0.94. The product of G-SUVmax and ADCmin did not improve diagnostic efficiency (**Figure 3**, **Table 4**).

### The Correlations Between Ki-67 Index and Tumor Size as well as Imaging Parameters: G-SUVmax, F-SUVmax, and ADCmin

The Ki-67 index, as a tumor proliferation marker, is closely correlated with the degree of malignancy in tumors. It is also an important factor in the WHO pNEN grading system. In this study, F-SUVmax showed a moderate positive correlation with the Ki-67 index (r = 0.582, *p* < 0.001). In contrast, G-SUVmax and ADCmin had a moderate negative correlation with the Ki-67 index (G-SUVmax and Ki-67, r=-0.647, *p* < 0.001; ADCmin and Ki-67, r=-0.503, *p* < 0.01).

**Figure 4:**
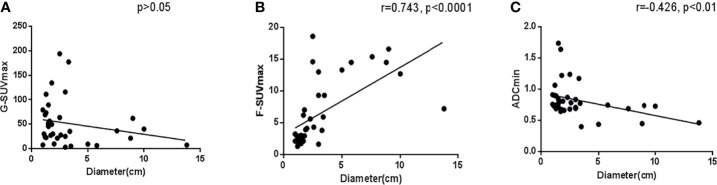
Correlation analysis of various imaging parameters and the maximum tumor diameter.

Tumor size is often considered an important factor that affects the accuracy of lesion evaluation in imaging analysis. In this study, in tumors with a diameter > 1 cm, there was no clear correlation between G-SUVmax and tumor maximum diameter (*p* > 0.05). However, we found that the maximum tumor diameter was highly positively correlated with F-SUVmax (r = 0.743, *p* < 0.001), but moderately negatively correlated with ADCmin (r = -0.426, *p* < 0.05). It demonstrated that in semiquantitative imaging analysis, the tumor size has some correlation with F-SUVmax and ADCmin but has little relationship with G-SUVmax (**Figure 4**).

### Features of Primary Pancreatic Lesions

**Figure 5:**
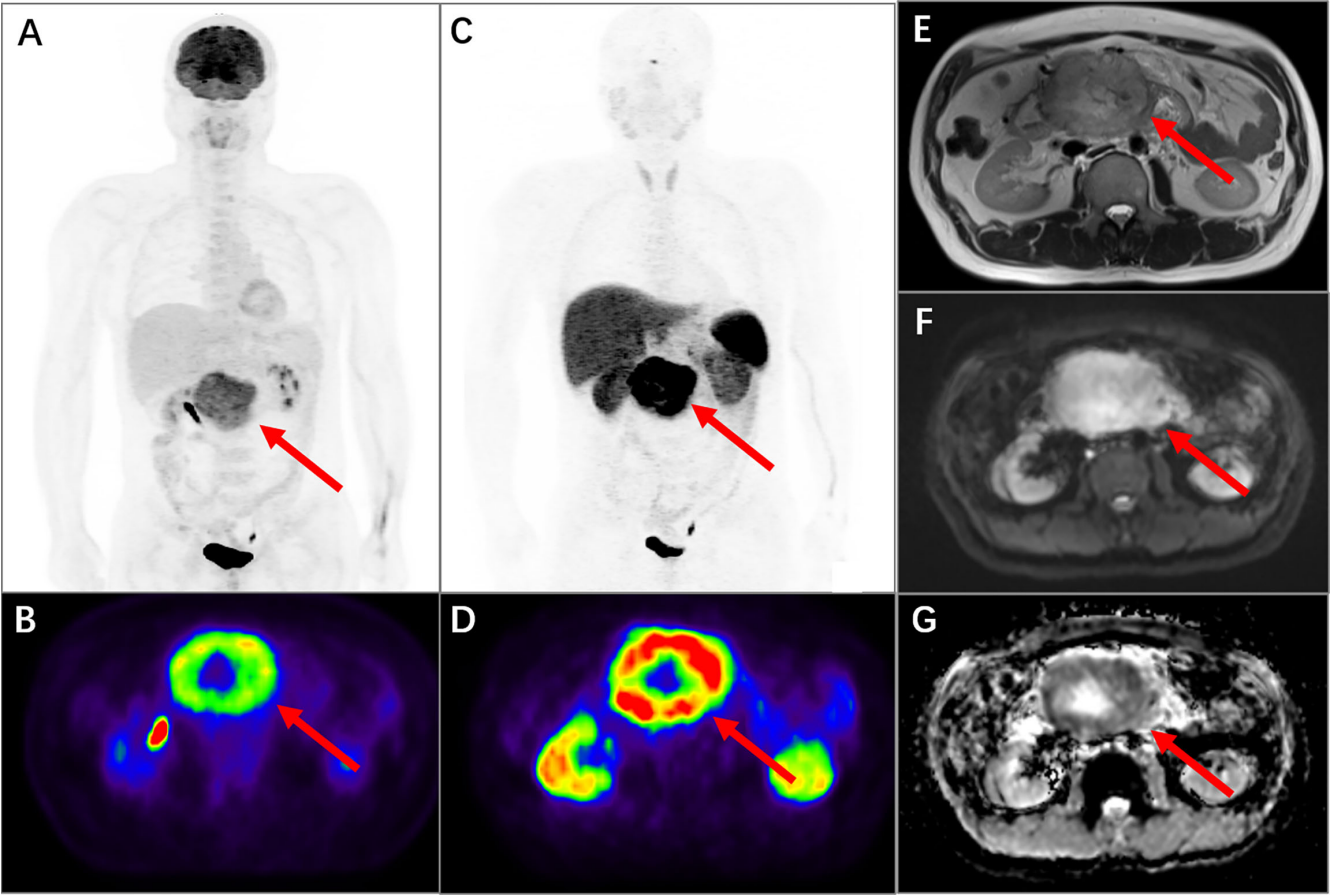
PET/MR images of a 48-year-old man with pancreatic neuroendocrine neoplasm grading G2. In ^18^F-FDG PET MIP (**A**, arrow) and transversal (**B**, arrow) images, it presented an intense uptake in the upper abdomen with SUVmax of 12.7. In ^68^Ga-DOTATATE PET MIP (**C**, arrow) and transversal (**D**, arrow) images, it also presented an obvious uptake with SUVmax of 40.2. The mass was located at the head of the pancreas with diameters of 10.0 × 8.0 × 7.5 cm and presented in the MR sequences of cross-sectional T2-weighted HASTE (**E**, arrow), cross-sectional DWI sequence (**F**, arrow), and ADC map (**G**, arrow). The ADCmin of the mass was 0.73.

Focusing on primary pancreatic lesions only, ^68^Ga-DOTATATE PET/MR still had outstanding performance in diagnosis and grading. There were 22 primary pancreatic lesions included in this study, including 15 patients grading G1, 4 patients grading G2, and 3 patients grading G3 (**Figure 5**).

In terms of diagnosis, the detection rates of ^68^Ga-DOTATATE PET alone were 14/15 in G1, 3/4 in G2, and 1/3 in G3, respectively. The detection rates of ^18^F-FDG PET alone were 7/15 in G1, 4/4 in G2, and 3/3 in G3, respectively. The ^68^Ga-DOTATATE PET combined with ^18^F-FDG PET or ^68^Ga-DOTATATE PET/MR imaging both could detect all grade primary pancreatic lesions in this study.

In terms of grading, the G-SUVmax decreased gradually as the grading increased [G1: 52.60(36.33,79.11), G2: 30.81(12.59,56.48), G3: 5.2, 7.2, 35.2]. The F-SUVmax was lowest in G1 but similar between G2 and G3 [G1: 2.33(1.78,6.97), G2: 9.15(4.45,15.61), G3: 9.3, 7.2, 9.3]. The ADCmin was lowest in G3 but similar between G1 and G2 [G1: 0.77(0.69,1.06), G2: 0.73(0.68,0.84), G3: 0.40, 0.46, 0.83].

In the 22 primary pancreas NENs, the G-SUVmax was still the only index not related to diameters (*p > 0.05*). The F-SUVmax was highly positively related to diameters (r = 0.765, *p < 0.0001*), while the ADCmin was moderately negatively related to diameters (r = -0.449, *p < 0.05*).

## Discussion

Our study systematically evaluated the values of major imaging parameters of ^68^Ga-DOTATATE + ^18^F-FDG PET/MRI in the diagnosis and grading prediction of pancreatic neuroendocrine tumors. Our results provide evidence for the selection of appropriate imaging techniques for the evaluation of pancreatic neuroendocrine tumors.

Currently, ^68^Ga-DOTA-SSA PET imaging is the preferred imaging technique for evaluation of pNENs. Studies have shown that ^68^Ga-DOTA-SSA PET can identify more lesions than traditional CT and MRI. It is of great value in the localization of occult primary tumors and the discovery of micrometastases in patients with metastasis (33). Recently, more and more studies have shown that the lesion detection rate of ^68^Ga-DOTA-SSA PET is not affected by octreotide treatment. Instead, octreotide administration leads to decreased or equal normal tissue uptake and an increased tumor-to-background ratio (30–32). Therefore, we do not request withdrawal of octreotide therapy before the ^68^Ga-DOTATATE PET scan in our practice, which is convenient for scheduling the scans and for the patients.

In this study, although ^68^Ga-DOTA-SSA PET imaging showed a high overall lesion detection rate of 80.6% in pNENs, its diagnostic efficacy varied greatly among various pathological grades of tumors. The diagnostic efficacy of ^68^Ga-DOTA-SSA PET was high for G1 and G2 pNENs with lesion detection rates of 95% and 87.5%, respectively. However, the detection efficiency for G3 and above pNENs was reduced to 37.5%. Therefore, ^68^Ga-DOTA-SSA PET/CT imaging alone tends to cause missed diagnosis of high-grade pNENs. In this case, other imaging modes, such as ^18^F-FDG PET/CT or MR imaging need to be combined in clinical practice to compensate the drawbacks of ^68^Ga-DOTA-SSA PET.

Previously, ^18^F-FDG PET was considered to have a low positive detection rate for NENs, and thus it was not recommended as a routine examination modality for the evaluation of NETs. However, recent studies have proposed that ^18^F-FDG PET/CT can be used as a supplemental technique with ^68^Ga-DOTA-SSA PET/CT to overcome its shortcoming for high-grade NENs. Kayani et al. investigated the application of ^18^F-FDG + ^68^Ga-DOTATATE PET/CT in imaging diagnoses of NENs and found that the sensitivities of ^18^F-FDG PET/CT and ^68^Ga-DOTATATE PET/CT alone were 66% and 82%, respectively, while the diagnostic sensitivity of ^18^F-FDG PET/CT + ^68^Ga-DOTATATE PET/CT could be increased to 92% (21). In our study, we found similar results where ^18^F-FDG + ^68^Ga-DOTATATE PET/MR can differentiate low-grade and high-grade pNENs and improve the lesion detection rate to 100%.

In the prediction of tumor pathological grade, SUVmax is closely associated with tumor grading in ^68^Ga-DOTATATE and ^18^F-FDG PET/CT imaging where low-grade tumors have higher ^68^Ga-DOTATATE uptake, while high-grade tumors have higher ^18^F-FDG uptake (21). In low-grade NETs (G1/G2), the ^18^F-FDG uptake in G2 NETs is higher than that in G1 NETs. However, it remains controversial regarding the uptake of ^68^Ga-DOTATATE in low-grade NETs. It is generally believed that the ^68^Ga-DOTATATE uptake in G2 NETs is lower than that in G1 NETs. However, in some studies, the ^68^Ga-DOTATATE uptake is higher in G2 NETs than in G1 tumors (28). Further analysis found that the primary lesions included in the later often arise from a mix of different primary sites such as stomach, small intestine, rectum, and pancreas. Some studies showed that primary NET lesions located at different sites have a different ^68^Ga-DOTATATE uptake. For example, the ^68^Ga-DOTATATE uptake in pancreatic NENs is higher than that of other NENs that originated at other primary sites (18, 34). Therefore, we speculate that the imaging analysis of mixed multi-origin NENs may lead to different conclusions due to the existence of bias. Thus, this study only analyzed primary NENs that localized in the pancreas. Our results showed that the SUVmax of grade G2 pNENs was significantly lower than that of grade G1 NETs in ^68^Ga-DOTATATE PET/MR imaging.

Previous studies have shown that in functional MRI imaging, the diffusion-weighted imaging (DWI) sequence and its quantitative index-apparent diffusion coefficient (ADC value) may potentially be used to predict the pathological grade of NENs. It has been shown that the average value and the minimum value of ADC in high-grade NENs (G3) were significantly lower than those of low-grade NETs (G1 and G2). However, the MR instrument, field strength, and b value of the cases included in the previous studies were not the same (27). In this study, all data from different patients were calculated on the same PET/MR instrument with fixed b values (50 and 800 s/mm^2^). Our results showed that ADCmin had no significant statistical difference between G1 and G2 pNENs. Its value was mainly in distinguishing low-grade (G1/G2) and high-grade (G3) NENs. The diagnostic efficiency of ADCmin was not as good as that of SUVmax of the ^18^F-FDG and ^68^Ga-DOTATATE. ADCmin combined with SUVmax of the ^68^Ga-DOTATATE did not improve the diagnostic efficiency of ^68^Ga-DOTATATE PET in differentiating low-grade and advanced pNENs.

In this study, we also analyzed the applicability of using multimodal imaging to improve pathological predictions of pNENs. The G-SUVmax/F-SUVmax ratio in ^68^Ga-DOTATATE + ^18^F-FDG PET imaging (G-SUVmax/F-SUVmax) only slightly improved the diagnostic specificity in distinguishing G1 and G2 pNENs and in differentiating low-grade and high-grade pNENs compared with ^68^Ga-DOTATATE PET alone. The G-SUVmax*ADCmin product in ^68^Ga-DOTATATE + ^18^F-FDG PET/MR imaging (G-SUVmax * F-SUVmax) did not improve the efficiency in the diagnosis and grading of pNENs when compared to ^68^Ga-DOTATATE PET alone.

Among the imaging parameters included in this study, G-SUVmax had no significant correlation with lesion size, while F-SUVmax and ADCmin were both closely correlated with lesion size. These results indicate that ^68^Ga-DOTATATE PET imaging with G-SUVmax is more reliable than other parameters in clinical application because it is less affected by tumor size.

In summary, ^68^Ga-DOTATATE PET/MR performs well in the detection of pNENs and the prediction of the pathological grade of the tumors; therefore, it is not necessary to perform both ^68^Ga-DOTATATE + ^18^F-FDG PET/MR imaging for this purpose. Considering factors such as shortening the duration of examination and reducing the radiation dose that patients may receive as well as reducing medical costs, we recommend ^68^Ga-DOTATATE PET/MR as the first choice of imaging to assess pancreatic neuroendocrine tumors.

This study had some limitations. Firstly, the number of cases above grade G2 and G3 included in the study was relatively small, which may lead to the underestimation of the intergroup differences among various parameters. Secondly, the biological manifestations and treatment options of G3 NETs and NECs are markedly different. We were not able to analyze G3 NETs and NECs separately due to the limited number of cases that could be enrolled in the study. Future studies with more available cases are needed to further improve the analysis. There may be some imaging differences between low-grade NETs with and without metastasis, which is worthy of further exploration.

## Conclusion

In terms of diagnosis, ^68^Ga-DOTATATE PET/MR imaging alone or ^68^Ga-DOTATATE + ^18^F-FDG PET imaging can both effectively detect various grades of pNEN lesions. Regarding pathological grading, G-SUVmax in ^68^Ga-DOTATATE PET imaging is the most valuable imaging parameter for tumor grading, and it has the advantage of not being affected by lesion size. ^18^F-FDG uptake and ADC value on MR images cannot effectively improve the diagnostic efficiency of ^68^Ga-DOTATATE PET imaging in the prediction of pNEN pathological grading. ^68^Ga-DOTATATE PET/MR imaging, as an evaluation method of pancreatic neuroendocrine tumors, can provide a high lesion detection rate and accurately predict the pathological grade of the lesion, indicating that currently it is the most ideal imaging technique for pNENs.

## Data Availability Statement

The original contributions presented in the study are included in the article/supplementary material. Further inquiries can be directed to the corresponding authors.

## Ethics Statement

This study was approved by the ethics committee of our hospital (2020-52). The patients/participants provided their written informed consent to participate in this study.

## Author Contributions

JZ, RZ, JJ, and YZ designed the study. YJ and JJ provided potentially eligible patients and arranged clinical data. JZ, YP, HJ, and XH interpret images. JZ, RZ, JJ, and YZ analyzed the results. JZ and RZ wrote the manuscript. All authors contributed to the article and approved the submitted version.

## Funding

This work was supported by the National Natural Science Foundation of China (Nos. 81971644, 81671720, and 81471688) and the Foundation of National Facility for Translational Medicine (Shanghai) (No. TMSK-2020-116).

## Conflict of Interest

The authors declare that the research was conducted in the absence of any commercial or financial relationships that could be construed as a potential conflict of interest.

## Publisher’s Note

All claims expressed in this article are solely those of the authors and do not necessarily represent those of their affiliated organizations, or those of the publisher, the editors and the reviewers. Any product that may be evaluated in this article, or claim that may be made by its manufacturer, is not guaranteed or endorsed by the publisher.
